# Crystal structure of tetra­guanidinium [hexa­hydrogen hexa­arsenato(V)tetra­vanadate(V)] tetra­hydrate

**DOI:** 10.1107/S1600536814011349

**Published:** 2014-08-01

**Authors:** William T. A. Harrison

**Affiliations:** a Department of Chemistry, University of Aberdeen, Meston Walk, Aberdeen AB24 3UE, Scotland

**Keywords:** crystal structure, polyoxidometallate anion, vanadium, arsenic

## Abstract

The complete polyoxidometallate anion in the title compound, (CH_6_N_3_)_4_[H_6_V_4_As_6_O_30_]·4H_2_O, is generated by crystallographic inversion symmetry. The polyhedral building units are distorted VO_6_ octa­hedra and AsO_3_OH tetra­hedra. The VO_6_ units feature a short formal V=O double bond and are linked by a common edge. Two such V_2_O_6_ double octahedral units are linked by four isolated AsO_3_OH tetra­hedra to complete the anion, which features two inter­nal O—H⋯O hydrogen bonds. In the crystal, O—H⋯O hydrogen bonds between the polyoxidometallate anions generate (01-1) sheets. The sheets are connected by cation-to-cluster N—H⋯O hydrogen bonds, and cation-to-water N—H⋯O links also occur. The O atom of one of the water mol­ecules is disordered over two sites in a 0.703 (17):0.297 (17) ratio.

## Related literature   

For crystal structures containing the same type of anion accompanied by different counter-cations, see: Durif & Averbuch-Pouchot (1979 ▶); Nenoff *et al.* (1994 ▶); Bremner & Harrison (2002 ▶). The site symmetries of these anions include 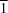 (as seen for the title compound) as well as 2/*m* and *mmm*.
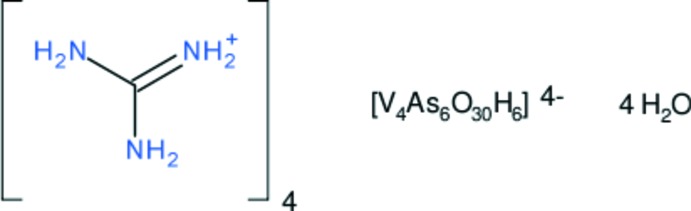


## Experimental   

### Crystal data  

(CH_6_N_3_)_4_[H_6_V_4_As_6_O_30_]·4H_2_O
*M* *_r_* = 1447.71
Triclinic, 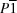

*a* = 10.0403 (5) Å
*b* = 11.0199 (6) Å
*c* = 11.9806 (6) Å
α = 114.892 (1)°
β = 94.696 (1)°
γ = 111.751 (1)°
*V* = 1071.39 (10) Å^3^
*Z* = 1
Mo *K*α radiation
μ = 5.56 mm^−1^
*T* = 293 K
0.30 × 0.20 × 0.20 mm

### Data collection  

Bruker SMART CCD diffractometer
Absorption correction: multi-scan (*SADABS*; Bruker, 1999 ▶) *T*_min_ = 0.287, *T*_max_ = 0.403
8711 measured reflections
4909 independent reflections
3655 reflections with *I* > 2σ(*I*)
*R*_int_ = 0.035

### Refinement  

*R*[*F* ^2^ > 2σ(*F* ^2^)] = 0.047
*wR*(*F* ^2^) = 0.129
*S* = 0.99
4909 reflections
271 parameters
H-atom parameters constrained
Δρ_max_ = 1.79 e Å^−3^
Δρ_min_ = −1.25 e Å^−3^

### 

Data collection: *SMART* (Bruker, 1999 ▶); cell refinement: *SAINT* (Bruker, 1999 ▶); data reduction: *SAINT*; program(s) used to solve structure: *SHELXS97* (Sheldrick, 2008 ▶); program(s) used to refine structure: *SHELXL97* (Sheldrick, 2008 ▶); molecular graphics: *ORTEP-3 for Windows* (Farrugia, 2012 ▶) and *ATOMS* (Dowty, 1999 ▶); software used to prepare material for publication: *SHELXL97*.

## Supplementary Material

Crystal structure: contains datablock(s) I, New_Global_Publ_Block. DOI: 10.1107/S1600536814011349/wm0004sup1.cif

Structure factors: contains datablock(s) I. DOI: 10.1107/S1600536814011349/wm0004Isup2.hkl

**Fig. 1.**
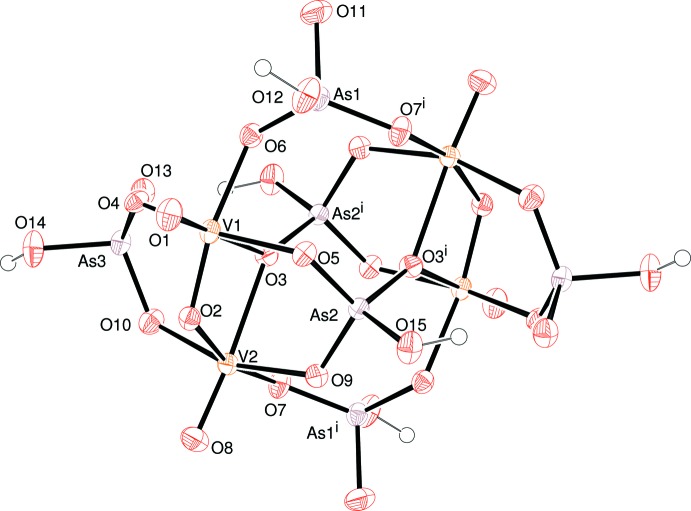
The molecular structure of the (V4As6O30H6)4- anion in the title compound showing 50% displacement ellipsoids. [Symmetry code: (i) –x, 1–y, 1–z.]

4 6 30 6 4− . DOI: 10.1107/S1600536814011349/wm0004fig1.tif
The mol­ecular structure of the (V_4_As_6_O_30_H_6_)^4−^ anion in the title compound showing 50% displacement ellipsoids. [Symmetry code: (i) –x, 1–y, 1–z.]

**Fig. 2.**
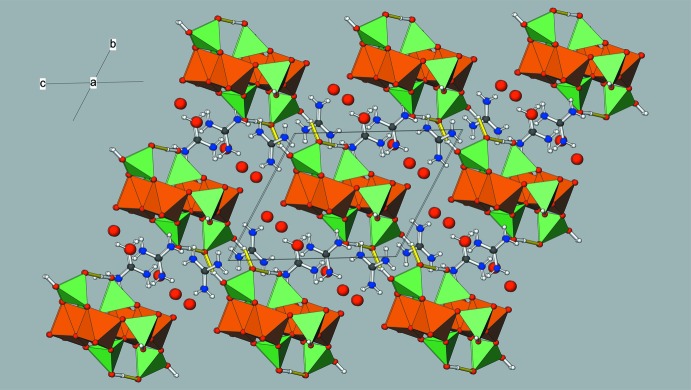
The packing of the title compound viewed down [100] with the anion shown in polyhedral representation (VO6 octahedra orange, AsO4 tetrahedra green). O—H···O hydrogen bonds within and between the anions are shown as yellow lines.

6 4 . DOI: 10.1107/S1600536814011349/wm0004fig2.tif
The packing of the title compound viewed down [100] with the anion shown in polyhedral representation (VO_6_ octa­hedra orange, AsO_4_ tetra­hedra green). O—H⋯O hydrogen bonds within and between the anions are shown as yellow lines.

CCDC reference: 1004306

Additional supporting information:  crystallographic information; 3D view; checkCIF report

## Figures and Tables

**Table 1:** Selected bond lengths (Å)

V1—O1	1.591 (4)
V1—O2	1.723 (4)
V1—O5	1.963 (4)
V1—O4	1.992 (4)
V1—O6	2.029 (4)
V1—O3	2.376 (4)
V2—O8	1.603 (5)
V2—O2	1.934 (4)
V2—O9	2.006 (4)
V2—O7	2.015 (4)
V2—O10	2.027 (4)
V2—O3	2.260 (4)

**Table 2:** Hydrogen-bond geometry (Å, °)

*D*—H⋯*A*	*D*—H	H⋯*A*	*D*⋯*A*	*D*—H⋯*A*
O12—H12⋯O4^i^	0.98	1.72	2.678 (5)	165
O14—H14⋯O13^ii^	0.96	1.66	2.579 (6)	158
O15—H15⋯O13^iii^	0.96	1.67	2.619 (6)	170
N1—H1*B*⋯O13^iv^	0.86	2.26	3.056 (8)	154
N1—H1*A*⋯O6^iv^	0.86	2.18	3.007 (7)	160
N2—H2*A*⋯O15^v^	0.86	2.20	3.042 (8)	167
N2—H2*B*⋯O11^iv^	0.86	2.44	3.210 (8)	150
N2—H2*B*⋯O1^vi^	0.86	2.47	3.040 (8)	125
N3—H3*A*⋯O9^v^	0.86	2.05	2.895 (7)	169
N3—H3*B*⋯O1*W*	0.86	2.20	2.984 (10)	152
N4—H4*A*⋯O12^vi^	0.86	2.30	3.089 (9)	152
N4—H4*B*⋯O10^vii^	0.86	2.48	3.230 (9)	146
N5—H5*A*⋯O2*WA* ^v^	0.86	2.14	2.959 (13)	160
N5—H5*B*⋯O7^vii^	0.86	2.19	2.963 (8)	150
N6—H6*B*⋯O12^vi^	0.86	2.37	3.140 (9)	150
N6—H6*B*⋯O5^vi^	0.86	2.45	3.022 (7)	125
N6—H6*A*⋯O2*WB* ^v^	0.86	2.01	2.78 (5)	148

Symmetry codes: (i) 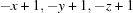; (ii) 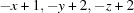; (iii) 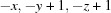; (iv) 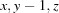; (v) 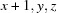; (vi) 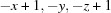; (vii) 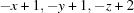.

## supplementary crystallographic information

### S1. Synthesis and crystallization

0.91 g of V_2_O_5_ and 0.90 g of (CN_3_H_6_)_2_CO_3_ were added to 10 ml of a 0.1 M H_3_AsO_4_ solution and placed in a Teflon-lined hydro­thermal vessel, which was heated to 423 K for 24 hours. After cooling to room temperature over several hours, solids were recovered by vacuum filtration to yield a few orange blocks of the title compound accompanied by an unidentified brown powder.

### S2. Refinement

The H atoms were located in different maps (O—H) or geometrically placed (N—H) and refined as riding atoms with *U*_iso_(H) = 1.2*U*_eq_(carrier). The water-molecule H atoms could not be located in the present experiment. One of the water molecule O atoms is disordered over two adjacent sites in a 0.703 (17):0.297 (17) ratio.

### Crystal data

**Table:**

(CH_6_N_3_)_4_[H_6_V_4_As_6_O_30_]·4H_2_O	*Z* = 1
*M**_r_* = 1447.71	*F*(000) = 704
Triclinic, *P*1	*D*_x_ = 2.244 Mg m^−^^3^
*a* = 10.0403 (5) Å	Mo *K*α radiation, λ = 0.70173 Å
*b* = 11.0199 (6) Å	Cell parameters from 3266 reflections
*c* = 11.9806 (6) Å	θ = 2.3–27.5°
α = 114.892 (1)°	µ = 5.56 mm^−^^1^
β = 94.696 (1)°	*T* = 293 K
γ = 111.751 (1)°	Block, orange
*V* = 1071.39 (10) Å^3^	0.30 × 0.20 × 0.20 mm

### Data collection

**Table:**

Bruker SMART CCD diffractometer	4909 independent reflections
Radiation source: fine-focus sealed tube	3655 reflections with *I* > 2σ(*I*)
Graphite monochromator	*R*_int_ = 0.035
ω scans	θ_max_ = 27.2°, θ_min_ = 1.9°
Absorption correction: multi-scan (*SADABS*; Bruker, 1999)	*h* = −13→13
*T*_min_ = 0.287, *T*_max_ = 0.403	*k* = −14→12
8711 measured reflections	*l* = −11→15

### Refinement

**Table:**

Refinement on *F*^2^	Primary atom site location: structure-invariant direct methods
Least-squares matrix: full	Secondary atom site location: difference Fourier map
*R*[*F*^2^ > 2σ(*F*^2^)] = 0.047	Hydrogen site location: mixed
*wR*(*F*^2^) = 0.129	H-atom parameters constrained
*S* = 0.99	*w* = 1/[σ^2^(*F*_o_^2^) + (0.0766*P*)^2^] where *P* = (*F*_o_^2^ + 2*F*_c_^2^)/3
4909 reflections	(Δ/σ)_max_ < 0.001
271 parameters	Δρ_max_ = 1.79 e Å^−^^3^
0 restraints	Δρ_min_ = −1.25 e Å^−^^3^

### Special details

**Table:**

Geometry. All e.s.d.'s (except the e.s.d. in the dihedral angle between two l.s. planes) are estimated using the full covariance matrix. The cell e.s.d.'s are taken into account individually in the estimation of e.s.d.'s in distances, angles and torsion angles; correlations between e.s.d.'s in cell parameters are only used when they are defined by crystal symmetry. An approximate (isotropic) treatment of cell e.s.d.'s is used for estimating e.s.d.'s involving l.s. planes.
Refinement. Refinement of *F*^2^ against ALL reflections. The weighted *R*-factor *wR* and goodness of fit *S* are based on *F*^2^, conventional *R*-factors *R* are based on *F*, with *F* set to zero for negative *F*^2^. The threshold expression of *F*^2^ > σ(*F*^2^) is used only for calculating *R*-factors(gt) *etc*. and is not relevant to the choice of reflections for refinement. *R*-factors based on *F*^2^ are statistically about twice as large as those based on *F*, and *R*- factors based on ALL data will be even larger.

### Fractional atomic coordinates and isotropic or equivalent isotropic displacement parameters (Å^2^)

**Table:**

	*x*	*y*	*z*	*U*_iso_*/*U*_eq_	Occ. (<1)
V1	0.26837 (10)	0.46285 (10)	0.57607 (9)	0.0176 (2)	
V2	0.05032 (10)	0.50770 (11)	0.74922 (9)	0.0193 (2)	
As1	0.26156 (6)	0.46227 (6)	0.30244 (6)	0.02044 (15)	
As2	−0.08161 (6)	0.30204 (6)	0.44111 (5)	0.01626 (14)	
As3	0.39072 (6)	0.77706 (6)	0.84192 (5)	0.01955 (15)	
O1	0.3661 (5)	0.3738 (5)	0.5506 (4)	0.0282 (10)	
O2	0.1882 (4)	0.4284 (4)	0.6880 (4)	0.0192 (8)	
O3	0.1265 (4)	0.6002 (4)	0.6171 (4)	0.0194 (8)	
O4	0.4243 (4)	0.6617 (4)	0.7138 (4)	0.0202 (8)	
O5	0.0880 (4)	0.3108 (4)	0.4329 (4)	0.0198 (8)	
O6	0.3215 (4)	0.5543 (4)	0.4605 (4)	0.0211 (8)	
O7	−0.0827 (4)	0.6107 (5)	0.7760 (4)	0.0254 (9)	
O8	0.0098 (5)	0.4435 (5)	0.8461 (4)	0.0328 (10)	
O9	−0.1059 (4)	0.3374 (4)	0.5863 (4)	0.0216 (8)	
O10	0.2301 (4)	0.7032 (4)	0.8748 (4)	0.0253 (9)	
O11	0.3741 (5)	0.5860 (6)	0.2582 (5)	0.0389 (12)	
O12	0.2976 (5)	0.3108 (5)	0.2403 (4)	0.0299 (10)	
H12	0.4043	0.3389	0.2643	0.036*	
O13	0.4138 (4)	0.9295 (4)	0.8315 (4)	0.0260 (9)	
O14	0.5335 (5)	0.8266 (5)	0.9655 (4)	0.0319 (10)	
H14	0.5560	0.9049	1.0518	0.038*	
O15	−0.2016 (5)	0.1152 (4)	0.3450 (4)	0.0290 (10)	
H15	−0.2710	0.1031	0.2766	0.035*	
C1	0.6359 (8)	−0.0343 (7)	0.5654 (7)	0.0336 (15)	
N1	0.5480 (7)	−0.1219 (6)	0.6055 (6)	0.0441 (16)	
H1A	0.5002	−0.2169	0.5551	0.053*	
H1B	0.5389	−0.0834	0.6818	0.053*	
N2	0.6494 (8)	−0.0929 (7)	0.4497 (7)	0.0530 (19)	
H2A	0.7044	−0.0363	0.4229	0.064*	
H2B	0.6033	−0.1882	0.4001	0.064*	
N3	0.7054 (8)	0.1105 (7)	0.6418 (7)	0.0510 (18)	
H3A	0.7607	0.1680	0.6159	0.061*	
H3B	0.6959	0.1482	0.7180	0.061*	
C2	0.9422 (9)	0.0768 (8)	0.8896 (7)	0.0406 (17)	
N4	0.8450 (8)	0.0292 (9)	0.9471 (8)	0.069 (2)	
H4A	0.7854	−0.0646	0.9131	0.082*	
H4B	0.8411	0.0919	1.0186	0.082*	
N5	1.0309 (9)	0.2208 (7)	0.9432 (7)	0.061 (2)	
H5A	1.0945	0.2540	0.9067	0.073*	
H5B	1.0254	0.2818	1.0147	0.073*	
N6	0.9483 (9)	−0.0180 (8)	0.7813 (7)	0.064 (2)	
H6A	1.0112	0.0134	0.7435	0.076*	
H6B	0.8894	−0.1120	0.7475	0.076*	
O1W	0.7341 (7)	0.3408 (9)	0.9023 (6)	0.074 (2)	
O2WA	0.2417 (12)	0.2587 (12)	0.7880 (10)	0.082 (4)*	0.703 (17)
O2WB	0.063 (5)	0.108 (5)	0.631 (5)	0.18 (2)*	0.297 (17)

### Atomic displacement parameters (Å^2^)

**Table:**

	*U*^11^	*U*^22^	*U*^33^	*U*^12^	*U*^13^	*U*^23^
V1	0.0164 (4)	0.0168 (5)	0.0200 (5)	0.0099 (3)	0.0038 (4)	0.0073 (4)
V2	0.0180 (4)	0.0218 (5)	0.0200 (5)	0.0095 (4)	0.0055 (4)	0.0113 (4)
As1	0.0179 (3)	0.0225 (3)	0.0212 (3)	0.0109 (2)	0.0055 (2)	0.0093 (2)
As2	0.0159 (3)	0.0134 (3)	0.0203 (3)	0.0069 (2)	0.0036 (2)	0.0089 (2)
As3	0.0180 (3)	0.0178 (3)	0.0190 (3)	0.0090 (2)	0.0025 (2)	0.0055 (2)
O1	0.026 (2)	0.029 (2)	0.034 (2)	0.0197 (18)	0.0072 (18)	0.0131 (19)
O2	0.0200 (18)	0.0169 (19)	0.022 (2)	0.0090 (15)	0.0038 (15)	0.0109 (16)
O3	0.0208 (18)	0.0184 (19)	0.025 (2)	0.0113 (15)	0.0075 (16)	0.0136 (17)
O4	0.0168 (18)	0.0189 (19)	0.0205 (19)	0.0072 (15)	0.0075 (15)	0.0062 (16)
O5	0.0183 (18)	0.0166 (19)	0.022 (2)	0.0088 (15)	0.0058 (15)	0.0067 (16)
O6	0.0197 (18)	0.0189 (19)	0.020 (2)	0.0072 (15)	0.0042 (15)	0.0069 (16)
O7	0.0196 (19)	0.027 (2)	0.027 (2)	0.0131 (16)	0.0031 (17)	0.0086 (18)
O8	0.032 (2)	0.044 (3)	0.033 (2)	0.018 (2)	0.013 (2)	0.027 (2)
O9	0.0207 (19)	0.023 (2)	0.022 (2)	0.0098 (16)	0.0057 (16)	0.0120 (17)
O10	0.022 (2)	0.025 (2)	0.024 (2)	0.0098 (16)	0.0073 (17)	0.0076 (18)
O11	0.027 (2)	0.046 (3)	0.052 (3)	0.010 (2)	0.014 (2)	0.035 (3)
O12	0.022 (2)	0.026 (2)	0.037 (2)	0.0171 (17)	0.0060 (18)	0.0053 (19)
O13	0.027 (2)	0.019 (2)	0.027 (2)	0.0086 (16)	0.0018 (17)	0.0084 (17)
O14	0.030 (2)	0.031 (2)	0.023 (2)	0.0181 (19)	−0.0040 (18)	0.0021 (19)
O15	0.029 (2)	0.0147 (19)	0.034 (2)	0.0065 (16)	−0.0027 (18)	0.0091 (18)
C1	0.039 (4)	0.026 (3)	0.049 (4)	0.018 (3)	0.018 (3)	0.026 (3)
N1	0.059 (4)	0.024 (3)	0.050 (4)	0.012 (3)	0.024 (3)	0.024 (3)
N2	0.082 (5)	0.036 (4)	0.052 (4)	0.026 (3)	0.042 (4)	0.028 (3)
N3	0.066 (4)	0.032 (3)	0.049 (4)	0.011 (3)	0.027 (3)	0.023 (3)
C2	0.048 (4)	0.030 (4)	0.037 (4)	0.019 (3)	0.007 (3)	0.011 (3)
N4	0.057 (5)	0.051 (5)	0.078 (6)	0.017 (4)	0.028 (4)	0.018 (4)
N5	0.090 (6)	0.031 (4)	0.045 (4)	0.016 (4)	0.032 (4)	0.012 (3)
N6	0.096 (6)	0.036 (4)	0.050 (4)	0.030 (4)	0.031 (4)	0.012 (3)
O1W	0.062 (4)	0.101 (5)	0.049 (4)	0.019 (4)	0.003 (3)	0.047 (4)

### Geometric parameters (Å, º)

**Table:**

V1—O1	1.591 (4)	O3—As2^i^	1.671 (4)
V1—O2	1.723 (4)	O7—As1^i^	1.665 (4)
V1—O5	1.963 (4)	O12—H12	0.9769
V1—O4	1.992 (4)	O14—H14	0.9637
V1—O6	2.029 (4)	O15—H15	0.9600
V1—O3	2.376 (4)	C1—N2	1.304 (9)
V2—O8	1.603 (5)	C1—N3	1.309 (9)
V2—O2	1.934 (4)	C1—N1	1.330 (8)
V2—O9	2.006 (4)	N1—H1A	0.8600
V2—O7	2.015 (4)	N1—H1B	0.8600
V2—O10	2.027 (4)	N2—H2A	0.8600
V2—O3	2.260 (4)	N2—H2B	0.8600
As1—O6	1.649 (4)	N3—H3A	0.8600
As1—O7^i^	1.665 (4)	N3—H3B	0.8600
As1—O12	1.708 (4)	C2—N6	1.304 (9)
As1—O11	1.726 (5)	C2—N5	1.313 (9)
As2—O3^i^	1.671 (4)	C2—N4	1.318 (10)
As2—O9	1.675 (4)	N4—H4A	0.8600
As2—O5	1.683 (4)	N4—H4B	0.8600
As2—O15	1.719 (4)	N5—H5A	0.8600
As3—O13	1.669 (4)	N5—H5B	0.8600
As3—O10	1.679 (4)	N6—H6A	0.8600
As3—O4	1.687 (4)	N6—H6B	0.8600
As3—O14	1.714 (4)		
			
O1—V1—O2	102.7 (2)	O13—As3—O4	109.0 (2)
O1—V1—O5	99.0 (2)	O10—As3—O4	117.33 (19)
O2—V1—O5	93.43 (17)	O13—As3—O14	108.5 (2)
O1—V1—O4	98.6 (2)	O10—As3—O14	107.1 (2)
O2—V1—O4	90.70 (17)	O4—As3—O14	101.8 (2)
O5—V1—O4	160.49 (18)	V1—O2—V2	119.8 (2)
O1—V1—O6	99.5 (2)	As2^i^—O3—V2	136.2 (2)
O2—V1—O6	157.65 (18)	As2^i^—O3—V1	137.1 (2)
O5—V1—O6	84.99 (16)	V2—O3—V1	86.10 (14)
O4—V1—O6	83.98 (16)	As3—O4—V1	124.0 (2)
O1—V1—O3	178.9 (2)	As2—O5—V1	121.6 (2)
O2—V1—O3	77.34 (16)	As1—O6—V1	125.2 (2)
O5—V1—O3	82.06 (15)	As1^i^—O7—V2	127.5 (2)
O4—V1—O3	80.26 (15)	As2—O9—V2	122.6 (2)
O6—V1—O3	80.37 (15)	As3—O10—V2	124.8 (2)
O8—V2—O2	99.5 (2)	As1—O12—H12	112.3
O8—V2—O9	100.3 (2)	As3—O14—H14	122.9
O2—V2—O9	87.28 (16)	As2—O15—H15	109.3
O8—V2—O7	96.9 (2)	N2—C1—N3	120.6 (6)
O2—V2—O7	163.55 (18)	N2—C1—N1	119.8 (6)
O9—V2—O7	88.82 (16)	N3—C1—N1	119.5 (7)
O8—V2—O10	97.8 (2)	C1—N1—H1A	120.0
O2—V2—O10	87.81 (16)	C1—N1—H1B	120.0
O9—V2—O10	161.83 (17)	H1A—N1—H1B	120.0
O7—V2—O10	90.97 (17)	C1—N2—H2A	120.0
O8—V2—O3	175.3 (2)	C1—N2—H2B	120.0
O2—V2—O3	76.60 (15)	H2A—N2—H2B	120.0
O9—V2—O3	82.29 (15)	C1—N3—H3A	120.0
O7—V2—O3	87.04 (16)	C1—N3—H3B	120.0
O10—V2—O3	79.55 (16)	H3A—N3—H3B	120.0
O6—As1—O7^i^	122.0 (2)	N6—C2—N5	121.2 (8)
O6—As1—O12	111.2 (2)	N6—C2—N4	120.1 (7)
O7^i^—As1—O12	102.3 (2)	N5—C2—N4	118.7 (7)
O6—As1—O11	103.8 (2)	C2—N4—H4A	120.0
O7^i^—As1—O11	110.7 (2)	C2—N4—H4B	120.0
O12—As1—O11	106.1 (2)	H4A—N4—H4B	120.0
O3^i^—As2—O9	114.15 (19)	C2—N5—H5A	120.0
O3^i^—As2—O5	112.86 (19)	C2—N5—H5B	120.0
O9—As2—O5	112.62 (19)	H5A—N5—H5B	120.0
O3^i^—As2—O15	108.9 (2)	C2—N6—H6A	120.0
O9—As2—O15	103.6 (2)	C2—N6—H6B	120.0
O5—As2—O15	103.63 (19)	H6A—N6—H6B	120.0
O13—As3—O10	112.3 (2)		
			
O1—V1—O2—V2	−178.0 (2)	O3^i^—As2—O5—V1	92.3 (3)
O5—V1—O2—V2	−77.9 (2)	O9—As2—O5—V1	−38.8 (3)
O4—V1—O2—V2	83.0 (2)	O15—As2—O5—V1	−150.1 (3)
O6—V1—O2—V2	7.3 (6)	O1—V1—O5—As2	152.3 (3)
O3—V1—O2—V2	3.14 (19)	O2—V1—O5—As2	48.8 (3)
O8—V2—O2—V1	179.4 (2)	O4—V1—O5—As2	−53.1 (6)
O9—V2—O2—V1	79.4 (2)	O6—V1—O5—As2	−108.9 (3)
O7—V2—O2—V1	2.9 (7)	O3—V1—O5—As2	−27.9 (2)
O10—V2—O2—V1	−83.1 (2)	O7^i^—As1—O6—V1	67.3 (3)
O3—V2—O2—V1	−3.3 (2)	O12—As1—O6—V1	−53.5 (3)
O8—V2—O3—As2^i^	−152 (2)	O11—As1—O6—V1	−167.1 (3)
O2—V2—O3—As2^i^	173.8 (3)	O1—V1—O6—As1	73.3 (3)
O9—V2—O3—As2^i^	84.8 (3)	O2—V1—O6—As1	−111.9 (4)
O7—V2—O3—As2^i^	−4.4 (3)	O5—V1—O6—As1	−25.0 (3)
O10—V2—O3—As2^i^	−95.9 (3)	O4—V1—O6—As1	171.1 (3)
O8—V2—O3—V1	37 (3)	O3—V1—O6—As1	−107.8 (3)
O2—V2—O3—V1	2.09 (13)	O8—V2—O7—As1^i^	−88.3 (3)
O9—V2—O3—V1	−86.94 (14)	O2—V2—O7—As1^i^	88.2 (6)
O7—V2—O3—V1	−176.15 (14)	O9—V2—O7—As1^i^	12.0 (3)
O10—V2—O3—V1	92.32 (15)	O10—V2—O7—As1^i^	173.8 (3)
O1—V1—O3—As2^i^	91 (11)	O3—V2—O7—As1^i^	94.3 (3)
O2—V1—O3—As2^i^	−174.0 (3)	O3^i^—As2—O9—V2	−85.4 (3)
O5—V1—O3—As2^i^	−78.6 (3)	O5—As2—O9—V2	45.0 (3)
O4—V1—O3—As2^i^	93.1 (3)	O15—As2—O9—V2	156.3 (2)
O6—V1—O3—As2^i^	7.6 (3)	O8—V2—O9—As2	−153.2 (3)
O1—V1—O3—V2	−97 (10)	O2—V2—O9—As2	−54.0 (3)
O2—V1—O3—V2	−2.33 (14)	O7—V2—O9—As2	110.0 (3)
O5—V1—O3—V2	93.02 (15)	O10—V2—O9—As2	20.5 (7)
O4—V1—O3—V2	−95.27 (15)	O3—V2—O9—As2	22.8 (2)
O6—V1—O3—V2	179.25 (15)	O13—As3—O10—V2	109.2 (3)
O13—As3—O4—V1	−112.7 (3)	O4—As3—O10—V2	−18.1 (4)
O10—As3—O4—V1	16.3 (4)	O14—As3—O10—V2	−131.8 (3)
O14—As3—O4—V1	132.8 (3)	O8—V2—O10—As3	137.6 (3)
O1—V1—O4—As3	−142.3 (3)	O2—V2—O10—As3	38.3 (3)
O2—V1—O4—As3	−39.3 (3)	O9—V2—O10—As3	−36.1 (7)
O5—V1—O4—As3	63.0 (6)	O7—V2—O10—As3	−125.3 (3)
O6—V1—O4—As3	118.9 (3)	O3—V2—O10—As3	−38.5 (3)
O3—V1—O4—As3	37.7 (3)		

Symmetry code: (i) −*x*, −*y*+1, −*z*+1.

### Hydrogen-bond geometry (Å, º)

**Table:**

*D*—H···*A*	*D*—H	H···*A*	*D*···*A*	*D*—H···*A*
O12—H12···O4^ii^	0.98	1.72	2.678 (5)	165
O14—H14···O13^iii^	0.96	1.66	2.579 (6)	158
O15—H15···O13^i^	0.96	1.67	2.619 (6)	170
N1—H1*B*···O13^iv^	0.86	2.26	3.056 (8)	154
N1—H1*A*···O6^iv^	0.86	2.18	3.007 (7)	160
N2—H2*A*···O15^v^	0.86	2.20	3.042 (8)	167
N2—H2*B*···O11^iv^	0.86	2.44	3.210 (8)	150
N2—H2*B*···O1^vi^	0.86	2.47	3.040 (8)	125
N3—H3*A*···O9^v^	0.86	2.05	2.895 (7)	169
N3—H3*B*···O1*W*	0.86	2.20	2.984 (10)	152
N4—H4*A*···O12^vi^	0.86	2.30	3.089 (9)	152
N4—H4*B*···O10^vii^	0.86	2.48	3.230 (9)	146
N5—H5*A*···O2*WA*^v^	0.86	2.14	2.959 (13)	160
N5—H5*B*···O7^vii^	0.86	2.19	2.963 (8)	150
N6—H6*B*···O12^vi^	0.86	2.37	3.140 (9)	150
N6—H6*B*···O5^vi^	0.86	2.45	3.022 (7)	125
N6—H6*A*···O2*WB*^v^	0.86	2.01	2.78 (5)	148

Symmetry codes: (i) −*x*, −*y*+1, −*z*+1; (ii) −*x*+1, −*y*+1, −*z*+1; (iii) −*x*+1, −*y*+2, −*z*+2; (iv) *x*, *y*−1, *z*; (v) *x*+1, *y*, *z*; (vi) −*x*+1, −*y*, −*z*+1; (vii) −*x*+1, −*y*+1, −*z*+2.

## References

[bb1] Bremner, C. A. & Harrison, W. T. A. (2002). *Acta Cryst.* E**58**, m254–m256.

[bb2] Bruker (1999). *SMART*, *SAINT* and *SADABS* Bruker AXS Inc., Madison, Wisconsin, USA.

[bb3] Dowty, E. (1999). *ATOMS* Shape Software, Kingsport, Tennessee, USA.

[bb4] Durif, A. & Averbuch-Pouchot, M. T. (1979). *Acta Cryst.* B**35**, 1441–1444.

[bb5] Farrugia, L. J. (2012). *J. Appl. Cryst.* **45**, 849–854.

[bb6] Nenoff, T. M., Stucky, G. D. & Harrison, W. T. A. (1994). *Z. Kristallogr.* **209**, 892–898.

[bb7] Sheldrick, G. M. (2008). *Acta Cryst.* A**64**, 112–122. 10.1107/S0108767307043930 18156677

