# Supplementary figures and images for: Crystal structure of tetra­guanidinium [hexa­hydrogen hexa­arsenato(V)tetra­vanadate(V)] tetra­hydrate
Source: Acta Crystallogr Sect E Struct Rep Online. 2014 Aug 1;70(Pt 9):m305–6. doi: 10.1107/S1600536814011349 (PMC4186164; doi:10.1107/S1600536814011349)

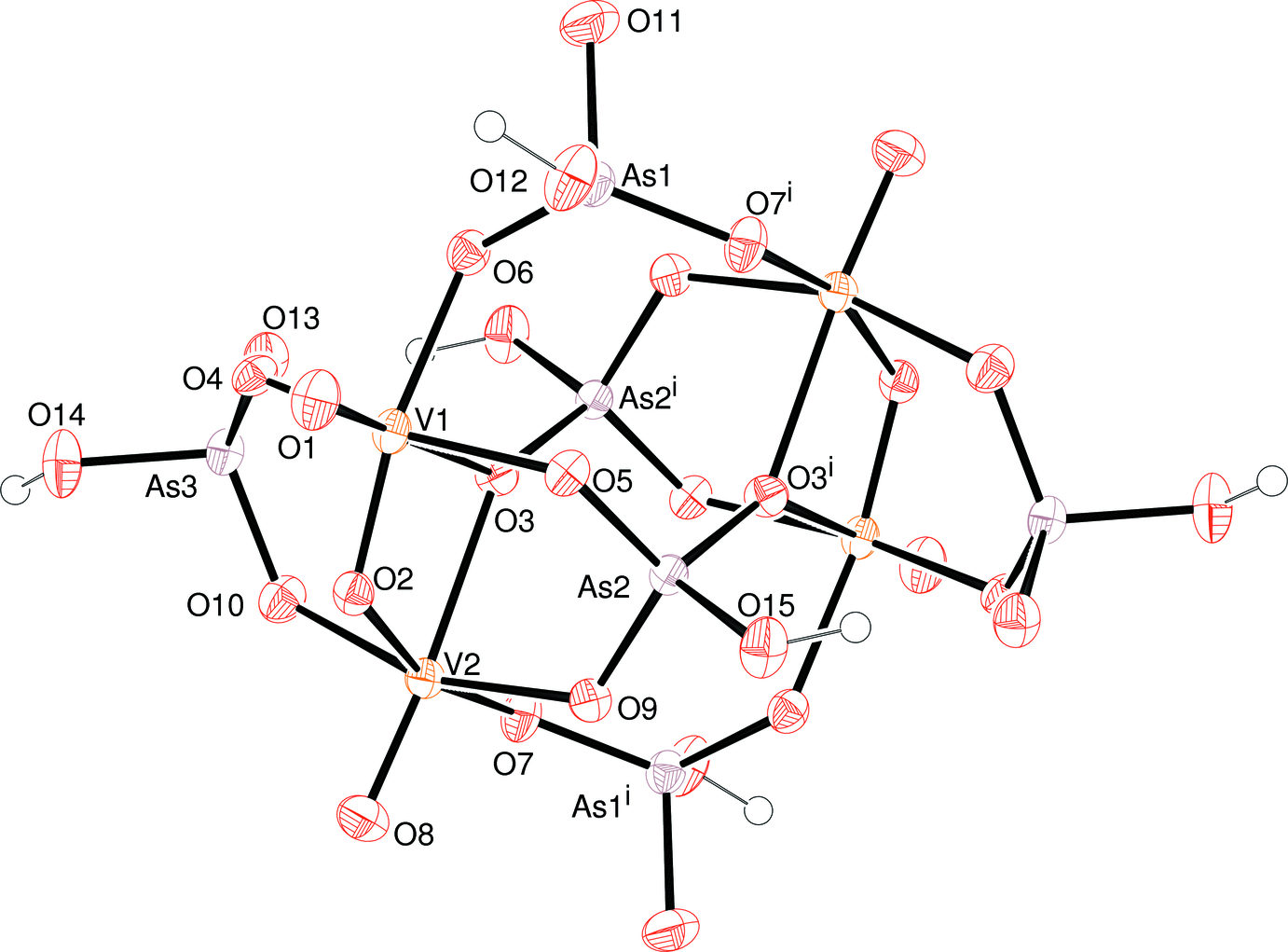

Supplement: Supplementary file 3 [file e-70-0m305-fig1.tif]

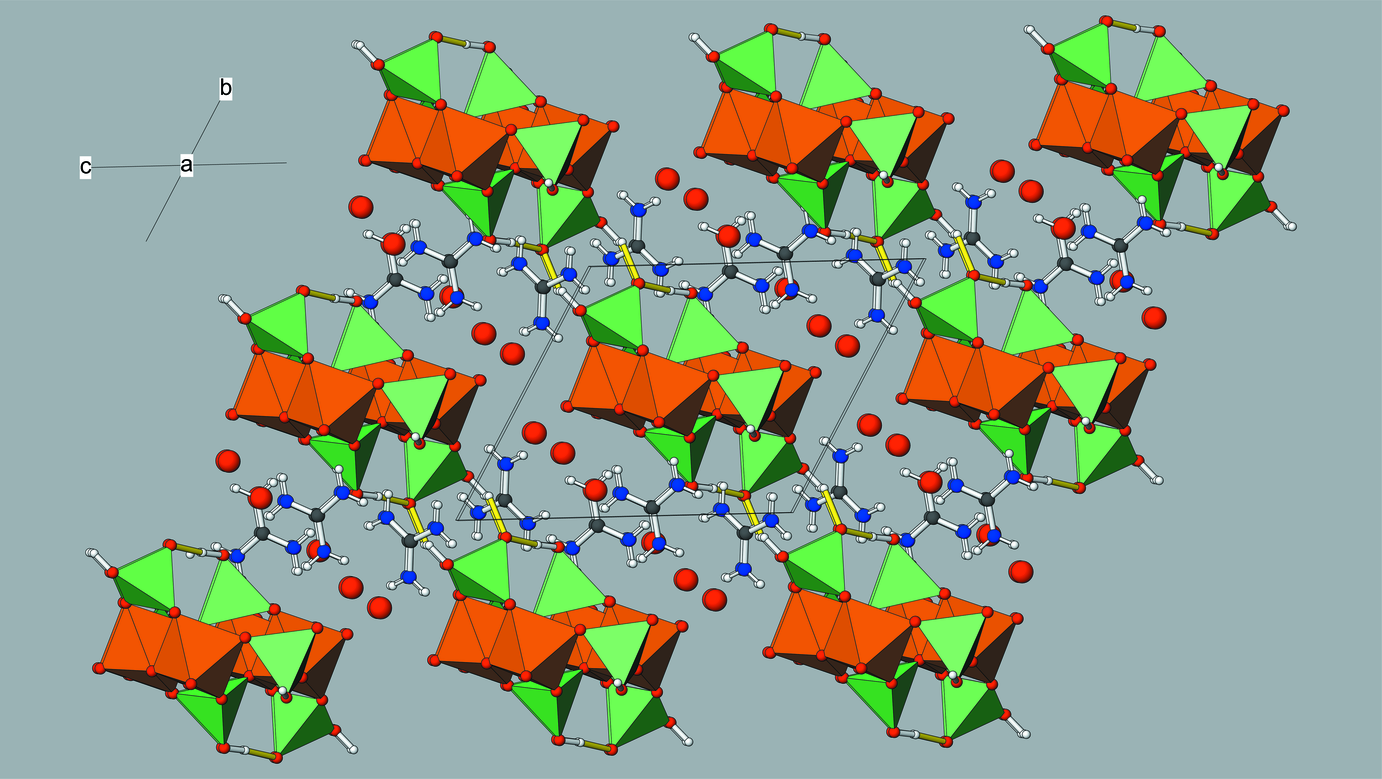

Supplement: Supplementary file 4 [file e-70-0m305-fig2.tif]
